# Paeonol ameliorates ferroptosis and inflammation in chondrocytes through AMPK/Nrf2/GPX4 pathway

**DOI:** 10.3389/fphar.2025.1526623

**Published:** 2025-03-07

**Authors:** Shuwei Gong, Shuang Lang, Xuesheng Jiang, Xiongfeng Li

**Affiliations:** ^1^ Department of Orthopedics, Huzhou Central Hospital, Fifth School of Clinical Medicine of Zhejiang Chinese Medical University, Huzhou, Zhejiang, China; ^2^ Huzhou Basic and Clinical Translation of Orthopedics Key Laboratory, Huzhou, Zhejiang, China; ^3^ Department of Traditional Chinese Medicine, Huzhou Central Hospital, Fifth School of Clinical Medicine of Zhejiang Chinese Medical University, Huzhou, Zhejiang, China

**Keywords:** paeonol, chondrocyte, ferroptosis, osteoarthritis, Nrf2

## Abstract

**Introduction:**

Chondrocyte ferroptosis is an important component of the pathogenesis of osteoarthritis. Paeonol, the main pharmacologically active ingredient of the Paeonia suffruticosa Andrews, is a natural radical scavenger with potent biological activities, including antioxidant, anti-inflammatory, and cartilage protection effects. However, the molecular mechanisms underlying its role in regulating chondrocytes ferroptosis remain unclear.

**Methods:**

To investigate the effect of paeonol on ferroptosis and inflammation of chondrocytes through interleukin-1β (IL-1β), the proliferation activity, lipid peroxidation level, endogenous antioxidant capacity, and mitochondrial membrane potential of chondrocytes were evaluated in detail. Intracellular ferrous ion concentration was detected by FerroOrange fluorescent probe staining. Western blotting and immunofluorescence staining were used to detect biomarker proteins of ferroptosis, inflammation, and AMPK/Nrf2/GPX4 signaling pathway proteins.

**Results:**

The results showed that paeonol significantly depressed IL-1β-induced ferroptosis and inflammation in chondrocytes. Specifically, paeonol protects cell viability, reduces lipid peroxidation damage, maintains mitochondrial function, and inhibits pro-ferroptosis and pro-inflammation biomarker proteins. In addition, the anti-inflammatory ability of paeonol was partially inhibited after the addition of ferroptosis agonist erastin, suggesting that paeonol protects against inflammatory injury in part by inhibiting ferroptosis. Further studies showed that paeonol activated AMPK phosphorylation and promoted Nrf2 nuclear translocation and Keap1 degradation. Finally, the AMPK-Nrf2-GPX4 signaling pathway was confirmed to be the underlying mechanism of paeonol against ferroptosis by the simultaneous use of the AMPK agonist and Nrf2 inhibitor.

**Conclusion:**

These results indicate that paeonol significantly inhibits IL-1β-induced ferroptosis and inflammation in chondrocytes, and the underlying mechanism of paeonol against ferroptosis is partly through the AMPK/Nrf2/GPX4 axis.

## Graphical Abstract

**Figure:**
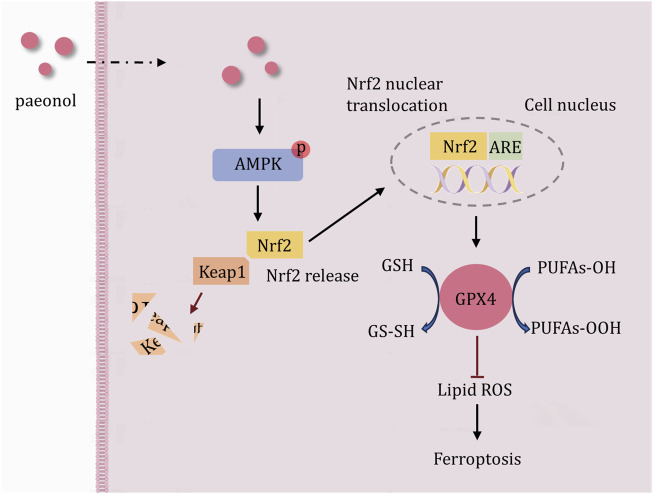
Schematic of the potential mechanism of paeonol against IL-1β-induced ferroptosis in chondrocytes. Paeonol promotes AMPK phosphorylation, followed by Keap1 degradation, Nrf2 nuclear translocation, maintenance of Nrf2 intracellular abundance, and finally activation of GPX4 to protect cells from excessive lipid peroxidation. Based on these findings, the AMPK-Nrf2-GPX4 signal channel is a potential mechanism by which paeonol inhibits ferroptosis in chondrocytes.

## Introduction

Knee osteoarthritis (KOA) is a common degenerative joint disease. Osteoarthritis affects approximately 7% of the world’s population (500 million) (Sharma, 2021; Srivastava, 2023). KOA is characterized by articular cartilage degeneration, joint space narrowing, subchondral bone sclerosis, and synovitis. Their clinical manifestations are chronic pain and dysfunction (Srivastava, 2023). Noteworthy, nearly 100 million people worldwide are disabled due to KOA, and knee osteoarthritis is the fourth leading cause of disability in the world (Zhu et al., 2023). The first-line management of KOA includes exercise therapy, health education, nonsteroidal anti-inflammatory drugs for patients with early-stage KOA, and knee arthroplasty for patients with end-stage KOA (Srivastava, 2023; Gibbs et al., 2023). In fact, nonsteroidal anti-inflammatory drugs not only have adverse effects such as gastric ulcers but also exhibit a dose ceiling effect (Arden et al., 2021). Total knee arthroplasty completely destroys the original cartilage of the knee joint. Moreover, long-term pain persists in 10%–34% of patients 3 months to 5 years after total knee arthroplasty (Sharma, 2021). Due to the high morbidity and treatment dilemma, it is imperative to explore new therapeutic targets based on the characteristic pathogenesis of KOA.

Ferroptosis is a newly programmed cell death characterized by uncontrolled lipid peroxidation catalyzed by divalent iron ions, eventually leading to cell death (Dixon and Olzmann, 2024). In the past 5 years, ferroptosis of chondrocytes has been confirmed to be closely related to the occurrence and progression of KOA (Sun et al., 2021; He et al., 2024). Specifically, chondrocytes isolated from KOA patients showed redox system disruption, manifested as decreased GPX4 content and intracellular iron overload (Miao et al., 2022). Subsequently, ferroptosis-induced chondrocyte metabolic imbalance in articular cartilage exacerbates the degradation of the extracellular matrix, including reduced glycosaminoglycan (GAG) and collagen II (Col-II), key cartilage components, accelerating the progression of KOA (Miao et al., 2022; Lv et al., 2022). Therefore, inhibiting ferroptosis in chondrocytes represents a crucial therapeutic strategy for mitigating the progression of knee osteoarthritis.

Paeonol (Pae) is a natural antioxidant found in the root of *Paeonia suffruticosa*, a traditional Chinese medicine (Liu et al., 2021; Liu et al., 2017). It has a variety of biological activities, such as antioxidant, anti-aging, and anti-inflammatory effects (Wu et al., 2024). A previous study pointed out that paeonol alleviates excessive oxidative stress induced by high glucose *in vitro* through an antioxidant response involving SIRT3 (Liu et al., 2024). In addition, paeonol reduced LPS-induced inflammation in RAW264.7 macrophages (Xu et al., 2018). As mentioned above, cellular peroxidation and inflammation are the characteristics of KOA. Thus, Pae is an essential candidate for treating KOA. In a recent arthria-related study, Pae alleviated cartilage wear by inhibiting IL-1β-induced inflammation and oxidative stress in OA chondrocytes (Liu et al., 2017). However, the underlying therapeutic mechanism of this process remains largely unknown. Considering the critical therapeutic value of ferroptosis of chondrocytes in OA, the present study aims to investigate Pae’s ability to inhibit chondrocyte ferroptosis and the underlying mechanism.

## Result

### Paeonol protected chondrocytes against oxidative stress injury induced by IL-1β

**FIGURE 1:**
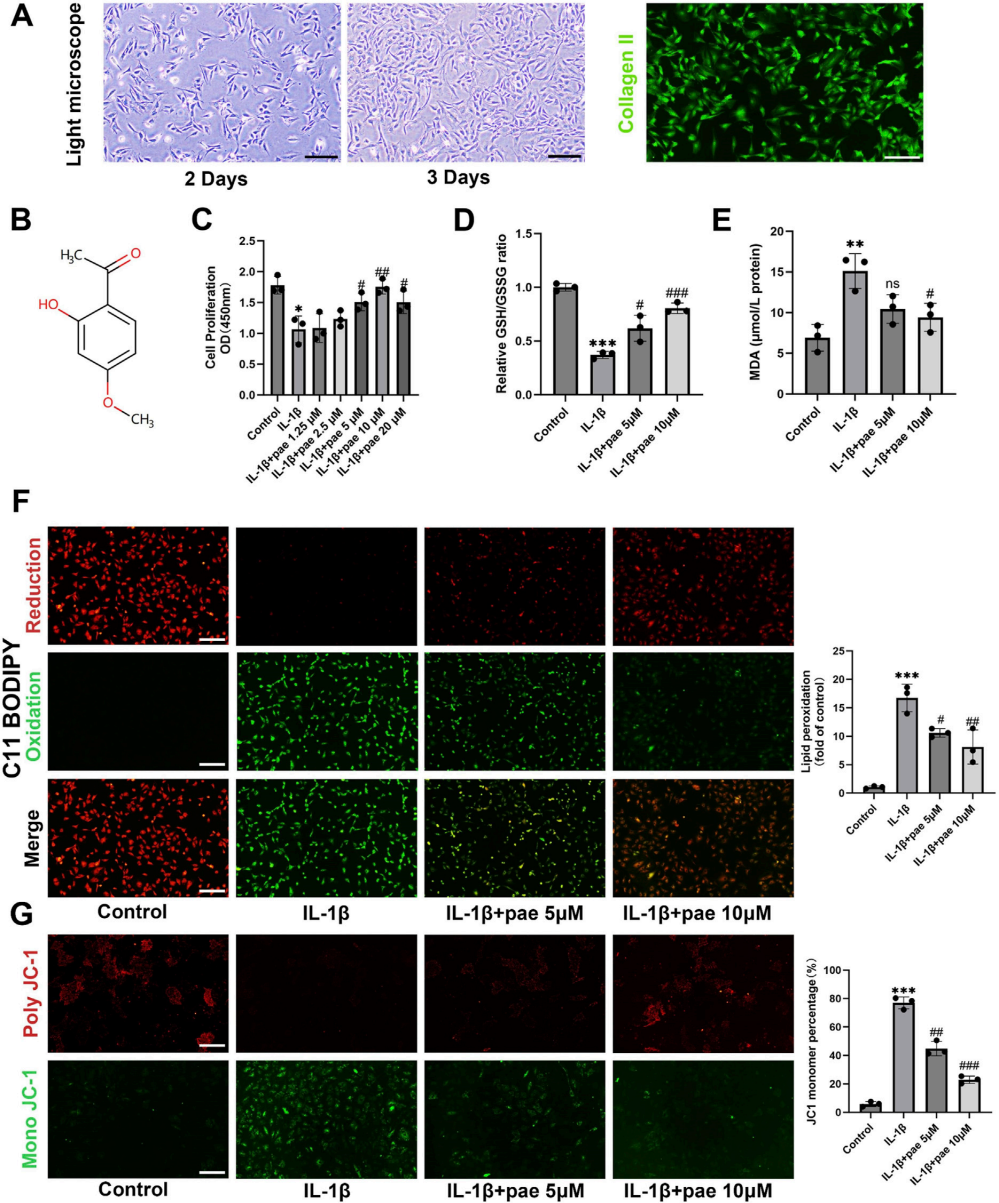
Paeonol protects chondrocyte viability and reduces lipid peroxidation under inflammatory conditions. **(A)** Morphology and collagen II immunofluorescence staining of primary chondrocytes; **(B)** The structure of paeonol; **(C)** Cell viability of rat chondrocytes treated with IL-1β and different concentrations of paeonol for 24 h; **(D)** The ratio of reduced glutathione to oxidized glutathione of rat chondrocytes treated with different intervention groups for 24 h; **(E)** Content of malondialdehyde, lipid peroxidation product, in rat chondrocytes treated with different intervention groups for 24 h. **(F)** Lipid ROS levels in chondrocytes treated with IL-1β and paeonol at different concentrations were detected by C11-BODIPY. **(G)** ΔΨm of chondrocytes in different treatment groups. The JC-1 polymer emits red fluorescence. The decrease in ΔΨm caused the JC-1 monomer to emit green fluorescence. All scale bar = 200 μm. Data are means ± SD. (n = 3). *P < *0.05*, **P < 0.01, ***P < *0.001* versus control group; ^*#*^ *P* < *0.05*, ^*##*^ *P* < *0.01,* ^*###*^ *P* < *0.001* versus IL-1β group; ns, not significant versus IL-1β group. Pae, Paeonol.

The extracted primary chondrocytes were triangular or fusiform after adhesion, and the morphology on the second and third day of culture was shown in Figure 1A. The purity of the extracted chondrocytes from cartilage was confirmed by immunofluorescence staining of collagen II (Figure 1A). The Structure of paeonol is shown in Figure 1B.

To verify the protective effect of paeonol on inflammatory chondrocytes, we established an inflammatory model of chondrocytes. Indeed, we found that the addition of IL-1β (10 ng/mL) significantly inhibited the activity of chondrocytes, while the activity of chondrocytes was partially rescued after treatment with concentrations of 5–20 μmol paeonol (Figure 1C). We further investigated the effects of paeonol on endogenous antioxidants under oxidative stress. Glutathione is a major endogenous antioxidant. The chondrocytes were subjected to oxidative stress under IL-1β stimulation, which showed a significant decrease in the GSH/GSSG ratio. It is worth noting that the GSH/GSSG reduction ratio was significantly elevated after paeonol treatment with 5 and 10 μM (Figure 1D). This suggests that paeonol can defend against inflammation-induced oxidative stress by enhancing the endogenous antioxidant system. The final outcome of ferroptosis is uncontrolled lipid peroxidation. We detected MDA, a marker of lipid peroxidation, and found that paeonol significantly inhibited the level of MDA increased by IL-1β (Figure 1E). C11-BODIPY581/591 lipid peroxidation fluorescence probe also confirmed the above conclusion, paeonol significantly reduced the green fluorescence, which represented the intensity of the oxidation (Figure 1F). Mitochondrial dysfunction is characterized by abnormal changes in mitochondrial membrane potential, which is one of the characteristics of ferroptosis. As shown in Figure 1G, IL-1β-induced chondrocyte mitochondrial membrane damage is manifested as an increase in the fragmentation of JC-1 aggregates (indicated by red fluorescence intensity) into JC-1 monomers (indicated by green fluorescence intensity). The decline in mitochondrial membrane potential was partially mitigated by paeonol administration (Figure 1G). This suggests that paeonol contributes to the preservation of membrane potential against IL-1β-induced oxidative damage. In brief, these data confirmed that paeonol has an exactly anti-lipid peroxidation effect on inflammatory chondrocytes.

### Paeonol protected chondrocytes against inflammation induced by IL-1β

**FIGURE 2:**
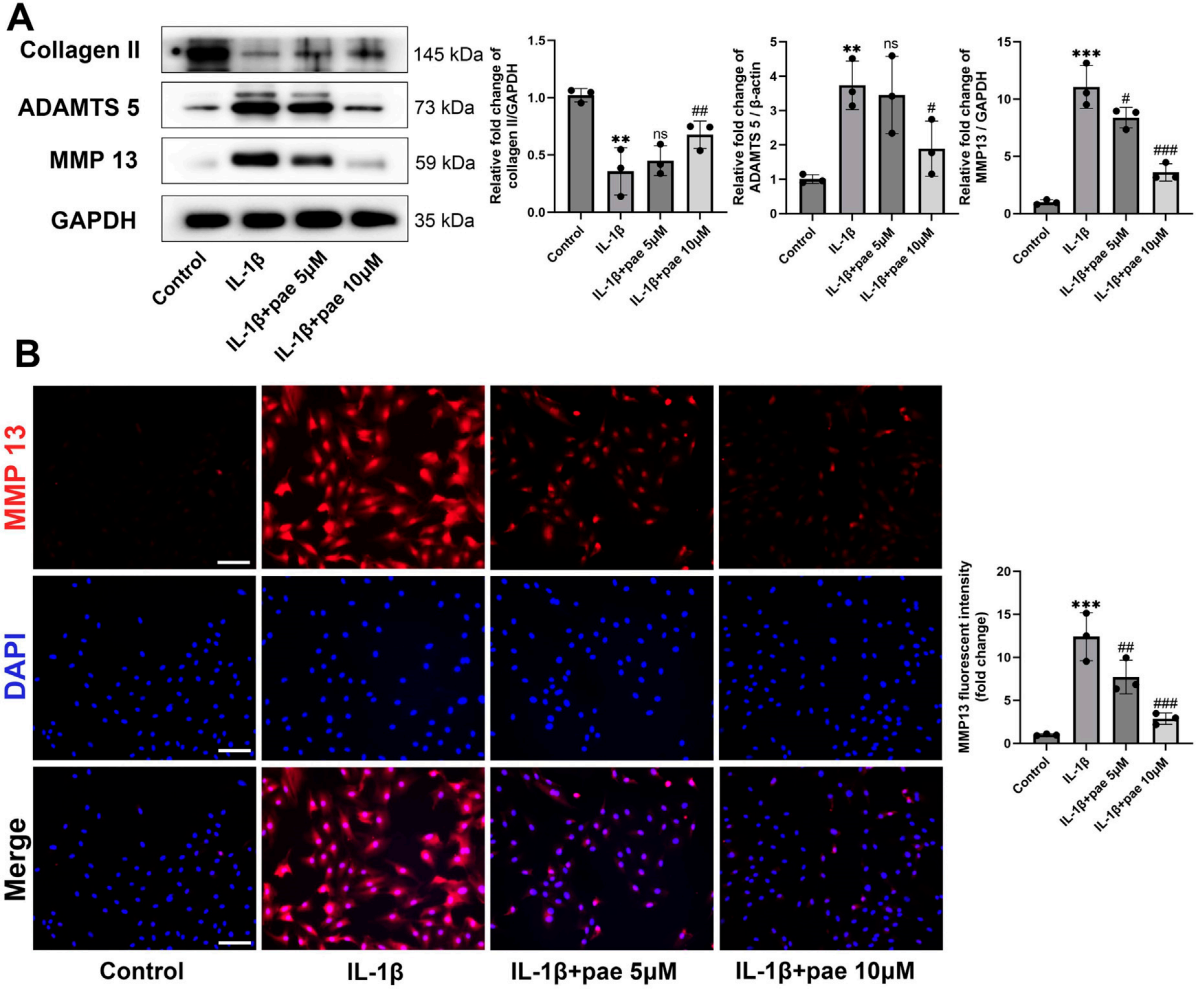
Paeonol alleviated the inflammatory phenotype of chondrocytes. **(A)** Western blot analysis for collagen Ⅱ and inflammation biomarkers ADAMTS 5, MMP 13 in chondrocyte treated with IL-1β and different concentrations of paeonol for 24 h. **(B)** Immunofluorescence staining for MMP 13 in chondrocytes treated with IL-1β and different concentrations of paeonol for 24 h. Scale bar = 100 μm. Data are means ± SD. (n = 3). **P < 0.01, ***P < *0.001* versus control group; ^*#*^ *P* < *0.05*, ^*##*^ *P* < *0.01,* ^*###*^ *P* < *0.001* versus IL-1β group; ns: not significant versus IL-1β group. Pae, Paeonol.

Meanwhile, paeonol significantly reversed IL-1β-induced elevation in the protein expression of inflammatory markers (Figure 2A). The results of Western Blots showed that the levels of ADAMTS5 and MMP13 were lower in the paeonol 5 μmol and 10 μmol groups than in the inflammatory IL-1β group (Figure 2A). Notably, paeonol 10 μmol was more potent than 5 μmol in inhibiting chondrocyte inflammation. Consistent with the above results, the addition of paeonol rescued IL-1β-inhibited collagen II levels in chondrocytes (Figure 2A). To further verify the anti-inflammatory effect of paeonol, immunofluorescence results of chondrocytes showed that the fluorescence intensity of MMP13 in the cells of the paeonol 5 μmol and 10 μmol groups was significantly lower than that of the IL-1β group (Figure 2B). These findings further demonstrate the significant role of paeonol in the anti-inflammatory levels of chondrocytes.

### Paeonol protected chondrocytes against ferroptosis induced by IL-1β

**FIGURE 3:**
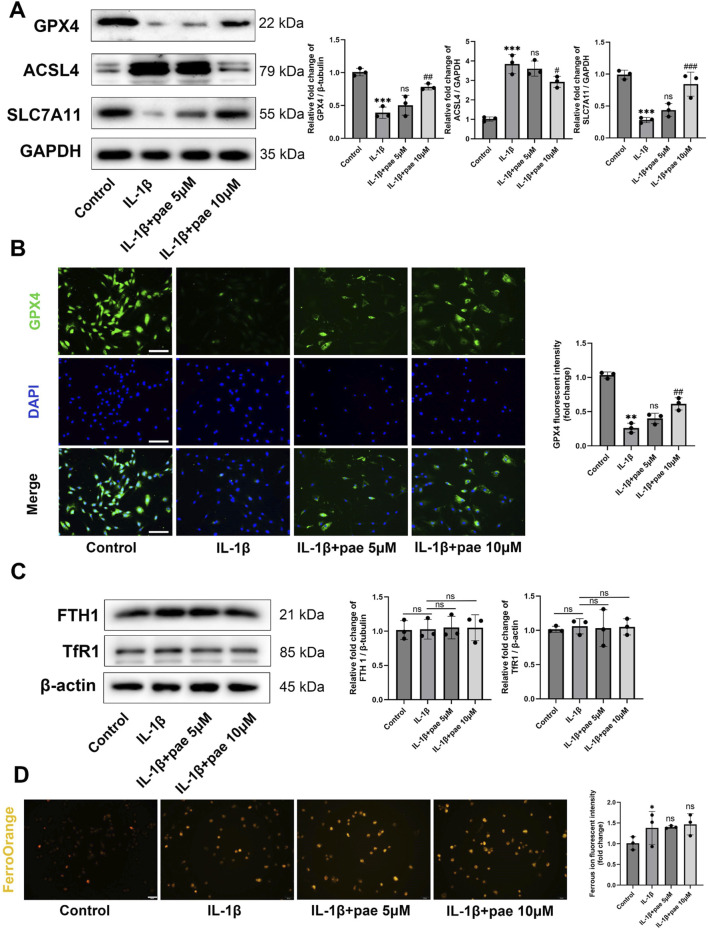
Paeonol alleviates ferroptosis in chondrocytes under inflammatory conditions but rarely regulates intracellular iron metabolism. **(A)** Western blot analysis for ferroptosis biomarkers GPX4, ACSL4, and SLC7A11 in chondrocyte treated with IL-1β and different concentrations of paeonol for 24 h. **(B)** Immunofluorescence staining for GPX4 in chondrocyte. Scale bar = 100 μm. **(C)** Western blot analysis for iron metabolism biomarkers FTH1 and, TfR1 in chondrocytes treated with IL-1β and different concentrations of paeonol for 24 h. **(D)** Ferrous ion levels in chondrocytes treated with IL-1β and paeonol at different concentrations were detected by a ferroOrange fluorescent probe. Scale bar = 100 μm. Data are means ± SD. (n = 3). *P < 0.05, **P < 0.01, ***P < *0.001* versus control group; ^*#*^ *P* < *0.05*, ^*##*^ *P* < *0.01,* ^*###*^ *P* < *0.001* versus IL-1β group; ns, not significant versus IL-1β group. Pae, Paeonol.

Next, we explored the role of paeonol in the regulation of ferroptosis in KOA chondrocytes. After IL-1β disposing, the protein levels of anti-ferroptosis markers GPX4 and SCL7A11 were significantly decreased, and the ferroptosis marker ACSL4 was significantly increased. These partial results confirm that ferroptosis is a pathological feature of chondrocytes under inflammatory conditions, which is consistent with the results of the previous study. More importantly, paeonol significantly reversed the decreased expression of GPX4, SCL7A11 and increased expression of ACSL4 induced by IL-1β (Figure 3A). Endogenous ferroptosis is accomplished by directly inhibiting the GPX4-related anti-lipid peroxidation system by the compounds RSL3, ML210, or FIN56. Immunohistochemical staining of GPX4 in chondrocytes confirmed that paeonol significantly enhanced the intensity of GPX4 inhibited by IL-1β. These results indicated that paeonol could regulate lipid peroxidation homeostasis to inhibit ferroptosis (Figure 3B).

In addition to excessive lipid peroxidation, iron overload is one of the features of ferroptosis. Ferritin heavy chain 1(FTH1) and Transferrin receptor protein 1 (TfR1) delicately regulate the storage and release of free iron in cells. However, our study rarely finds the effect of IL-1β and paeonol on iron metabolism in chondrocytes. This was reflected in the protein content of FTH1 and Tfr1 (Figure 3C). FerroOrange fluorescent probe staining showed that IL-1β slightly increased intracellular ferrous ion content, but paeonol did not showed significant regulatory effect on ferrous ion (Figure 3D). Therefore, we did not investigate the regulation of paeonol on cellular iron metabolism in the following study.

### Paeonol protects chondrocytes from inflammatory injury by inhibiting ferroptosis

**FIGURE 4:**
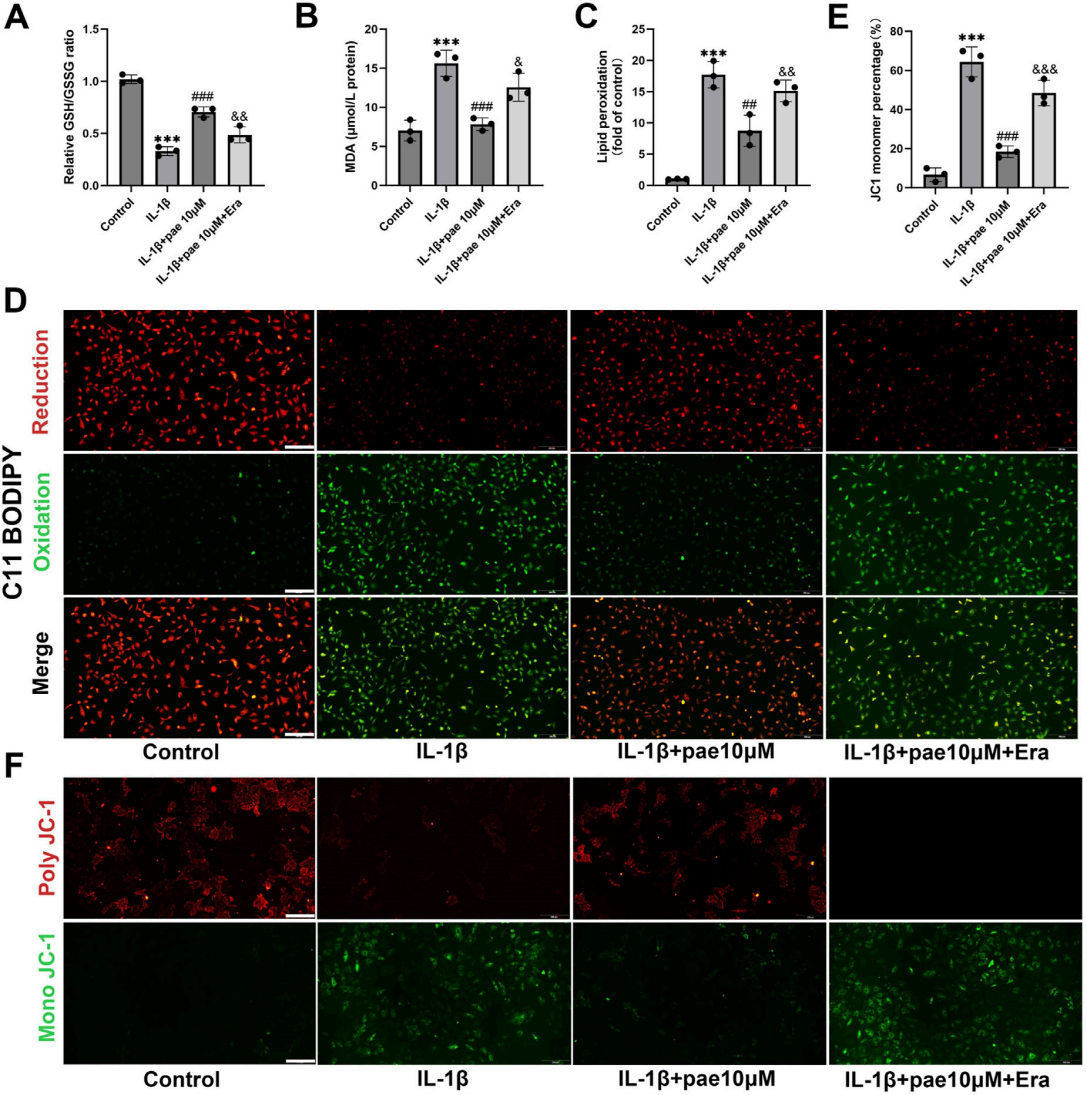
Paeonol inhibits IL-1β-induced oxidative damage in chondrocytes by inhibiting ferroptosis. **(A)** The ratio of reduced glutathione to oxidized glutathione of rat chondrocytes treated with IL-1β, IL-1β + paeonol 10 μM, and IL-1β + paeonol 10 μM + erastin 5 μM for 24 h; **(B)** Content of malondialdehyde, lipid peroxidation product, in chondrocytes treated with different intervention groups for 24 h. **(C, D)** Lipid ROS levels in chondrocytes treated with IL-1β and paeonol at different concentrations were detected by C11-BODIPY. **(E, F)** ΔΨm of chondrocytes in different treatment groups. The JC-1 polymer emits red fluorescence. The decrease in ΔΨm caused the JC-1 monomer to emit green fluorescence. All scale bar = 200 μm. Data are means ± SD. (n = 3). ***P < *0.001* versus control group; ^*##*^ *P* < *0.01,* ^*###*^ *P < 0.001* versus IL-1β group; *& P* < *0.05*, *&& P* < *0.01, &&& P* < *0.001* versus IL-1β+pae 10 μM group. Pae, Paeonol, Era, Erastin.

The above two studies confirmed the anti-inflammatory and anti-ferroptosis effects of paeonol in chondrocytes, but whether there is a crosstalk between these two protective effects remains unclear. In this part of the study, we added ferroptosis agonist Erastin to paeonol treatment. Our study also demonstrated that paeonol protected the endogenous antioxidant system, inhibited lipid peroxidation and maintained mitochondrial membrane potential in chondrocytes, as indicated by results of GSH/GSSG ratio, MDA content, C11 BODIPY staining, and JC1 staining (Figures 4A–F). Meanwhile, the above phenomena were partially reversed by Erastin (Figures 4A–F). This phenomenon is justified because Erastin acts as a ferroptosis agonist, and ferroptosis is characterized by elevated cellular lipid peroxidation.

**FIGURE 5:**
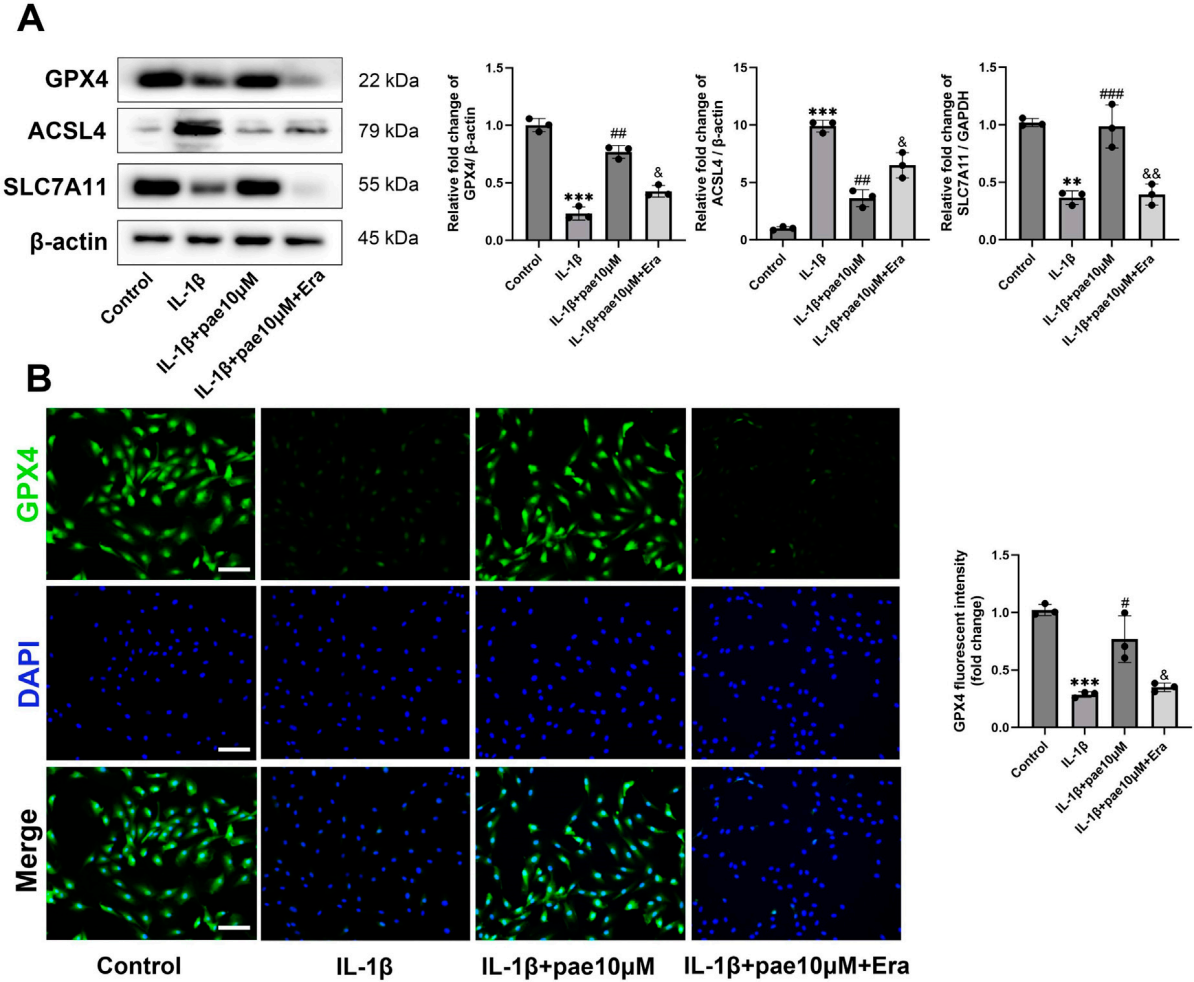
The anti-ferroptosis effect of paeonol was reversed by Erastin. **(A)** Western blot analysis for ferroptosis biomarkers GPX4, ACSL4, and SLC7A11 in chondrocyte treated with IL-1β, IL-1β + paeonol 10 μM, and IL-1β + paeonol 10 μM + erastin 5 μM for 24 h. **(B)** Immunofluorescence staining for GPX4 in chondrocyte. Scale bar = 100 μm. Data are means ± SD. (n = 3). **P < 0.01, ***P < *0.001* versus control group; ^*#*^ *P* < *0.05,* ^*##*^ *P* < *0.01,* ^*###*^ *P* < *0.001* versus IL-1β group; *&P* < *0.05*, *&&P* < *0.01* versus IL-1β+pae 10 μM group. Pae, Paeonol; Era, Erastin.

Western blot results further confirmed that the upregulation of GPX4, SLC7A11, and downregulation of ACSL4 by paeonol were partially reversed by Erastin (Figure 5A). Immunofluorescence staining of GPX4 also confirmed that paeonol significantly upregulated GPX4 expression in chondrocytes, which was inhibited by Erastin (Figure 5B).

**FIGURE 6:**
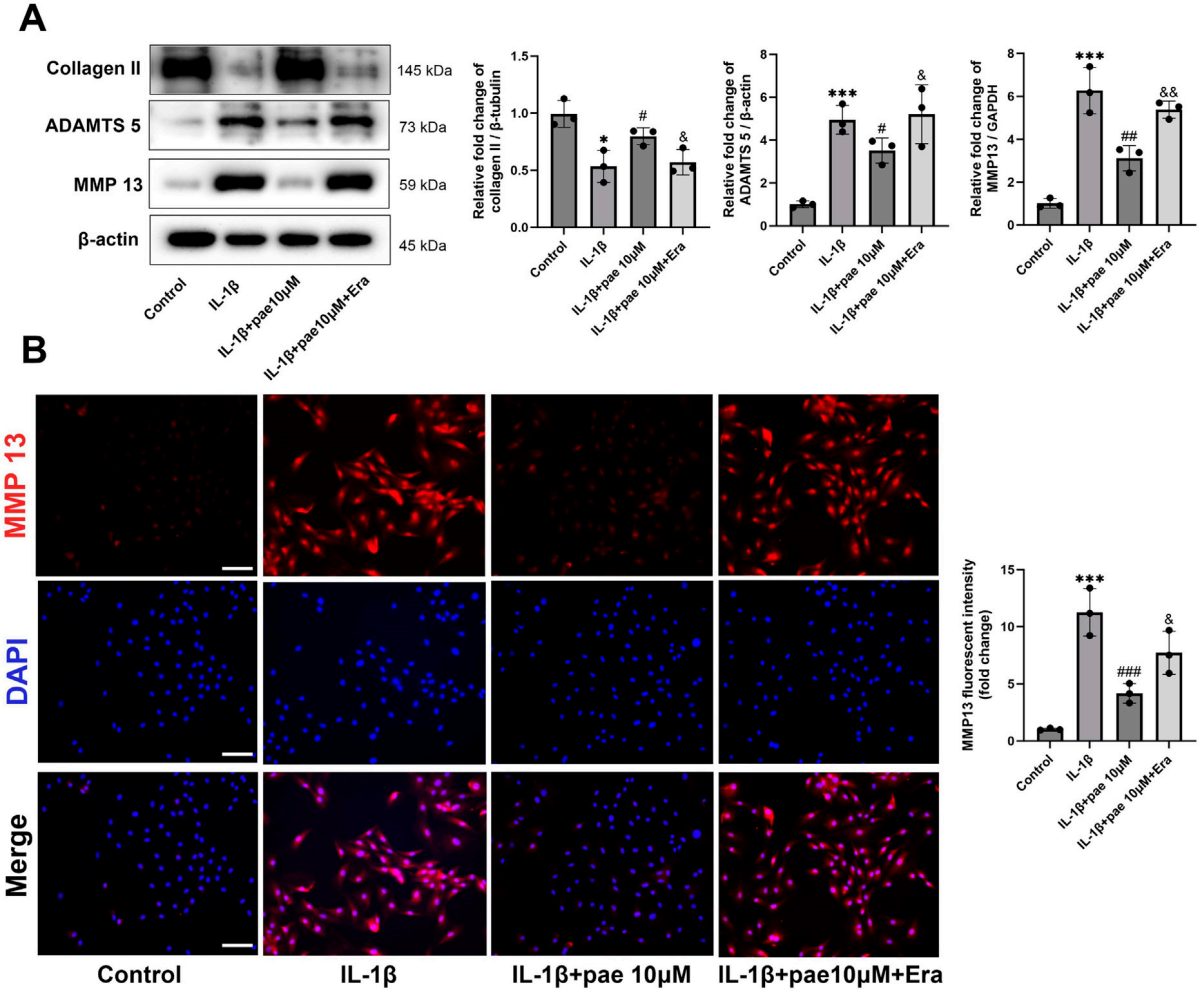
Paeonol alleviated the inflammatory phenotype of chondrocytes by inhibiting ferroptosis. **(A)** Western blot analysis for collagen Ⅱ and inflammation biomarkers ADAMTS 5, MMP 13 in chondrocyte treated with IL-1β, IL-1β + paeonol 10 μM, and IL-1β + paeonol 10 μM + erastin 5 μM for 24 h. **(B)** Immunofluorescence staining for MMP 13 in chondrocytes treated with different interventions for 24 h. Scale bar = 100 μm. Data are means ± SD. (n = 3). *P < 0.05, ***P < *0.001* versus control group; ^*#*^ *P* < *0.05*, ^*##*^ *P* < *0.01,* ^*###*^ *P* < *0.001* versus IL-1β group. *&P* < *0.05*, *&&P* < *0.01* versus IL-1β+pae 10 μM group. Pae, Paeonol; Era, Erastin.

Subsequently, Western Blots showed that paeonol-induced inhibition of chondrocyte inflammation was also partially reversed by Erastin (Figure 6A). Specifically, paeonol significantly reduced the protein levels of ADAMTS5 and MMP13 promoted by IL-1β, and this inhibitory effect was partially reversed by Erastin. This phenomenon was further confirmed by immunohistochemistry of MMP13 (Figure 6B). Notely, these results suggest that paeonol protects chondrocytes from IL-1β-induced inflammatory injury in part by inhibiting ferroptosis.

### Paeonol protected chondrocytes against ferroptosis through the AMPK-Nrf2-GPX4 signaling pathway

**FIGURE 7:**
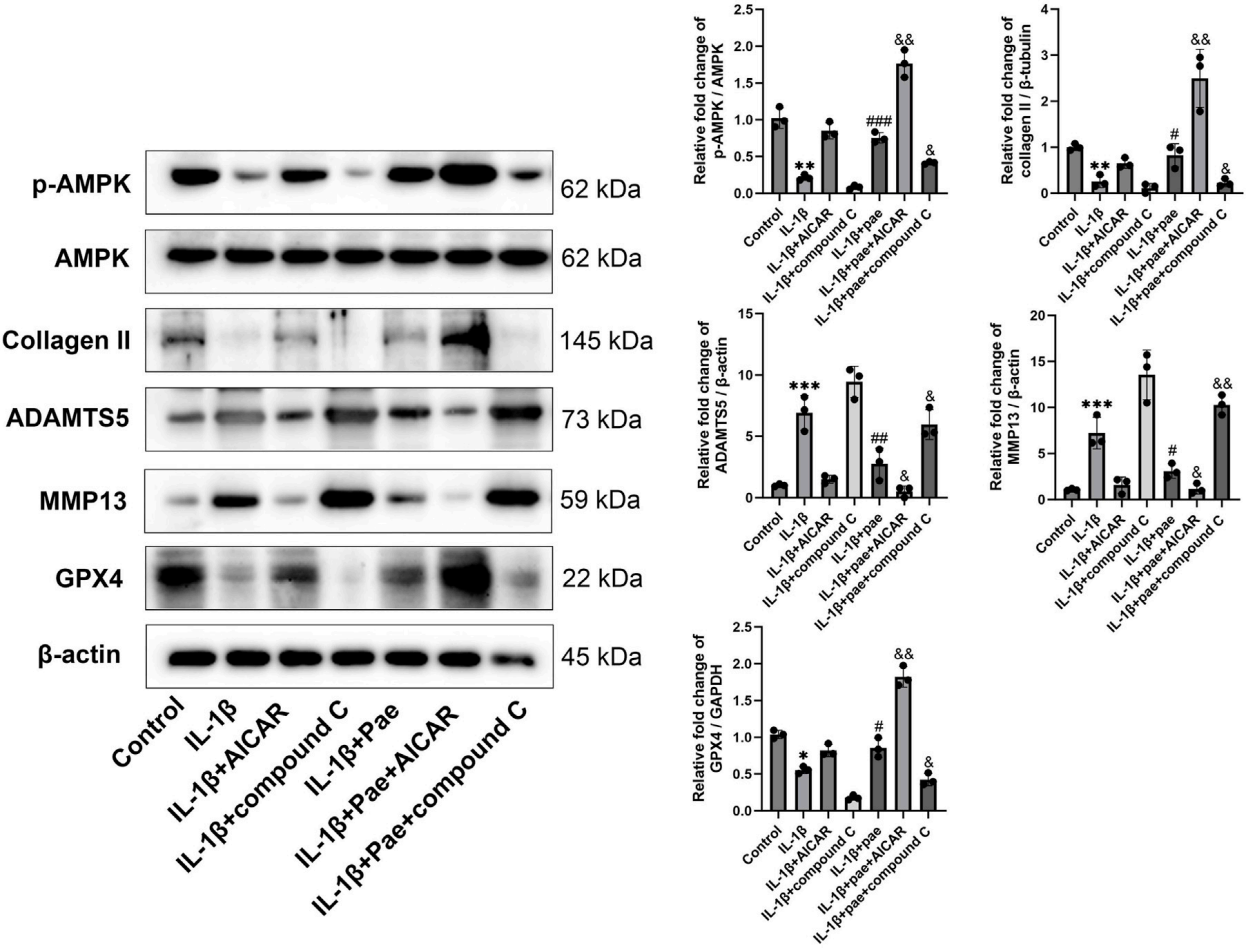
Paeonol inhibits inflammation and ferroptosis by inducing AMPK phosphorylation. Western blot of p-AMPK, AMPK, collagen II, inflammation biomarkers ADAMTS 5, MMP13, and ferroptosis biomarkers GPX4 in the presence of AMPK agonist AICAR and inhibitor compound C alone or paeonol plus AICAR and compound C. *P < 0.05, **P < 0.01, ***P < *0.001* versus control group; ^*#*^ *P* < *0.05*, ^*##*^ *P* < *0.01,* ^*###*^ *P* < *0.001* versus IL-1β group. *&P* < *0.05*, *&&P* < *0.01* versus IL-1β+pae 10 μM group. Data are means ± SD. (n = 3). Pae, Paeonol.

In the above study, we demonstrated that paeonol alleviated chondrocyte inflammation by inhibiting ferroptosis. However, the underlying mechanism by which paeonol inhibits ferroptosis in chondrocytes remains unclear. AMP-activated protein kinase (AMPK), an upstream regulator of antioxidative response, is closely related to cartilage degeneration. AMPK activation attenuated the protein levels of inflammatory markers IL-1β and TNF-α in chondrocytes. Moreover, emerging evidence suggests that AMPK has a role in inhibiting ferroptosis. Therefore, Compound C (a selective AMPK inhibitor) and AICAR (an AMPK agonist) were used for further experiments in this part of the study. Our results showed that paeonol significantly rescued AMPK phosphorylation, which was inhibited by IL-1β (Figure 7). Consistent with the above findings, paeonol significantly increased the protein level of COL II, decreased the inflammatory markers MMP13 and ADAMTS5, and increased the GPX4, a key regulator of ferroptosis, in chondrocytes. However, the addition of AICAR, an AMPK agonist, further enhanced the anti-inflammatory and anti-ferroptosis effects of paeonol, while the addition of Compound C, an AMPK inhibitor, reversed the regulatory effects of paeonol on inflammation and ferroptosis (Figure 7).

**FIGURE 8:**
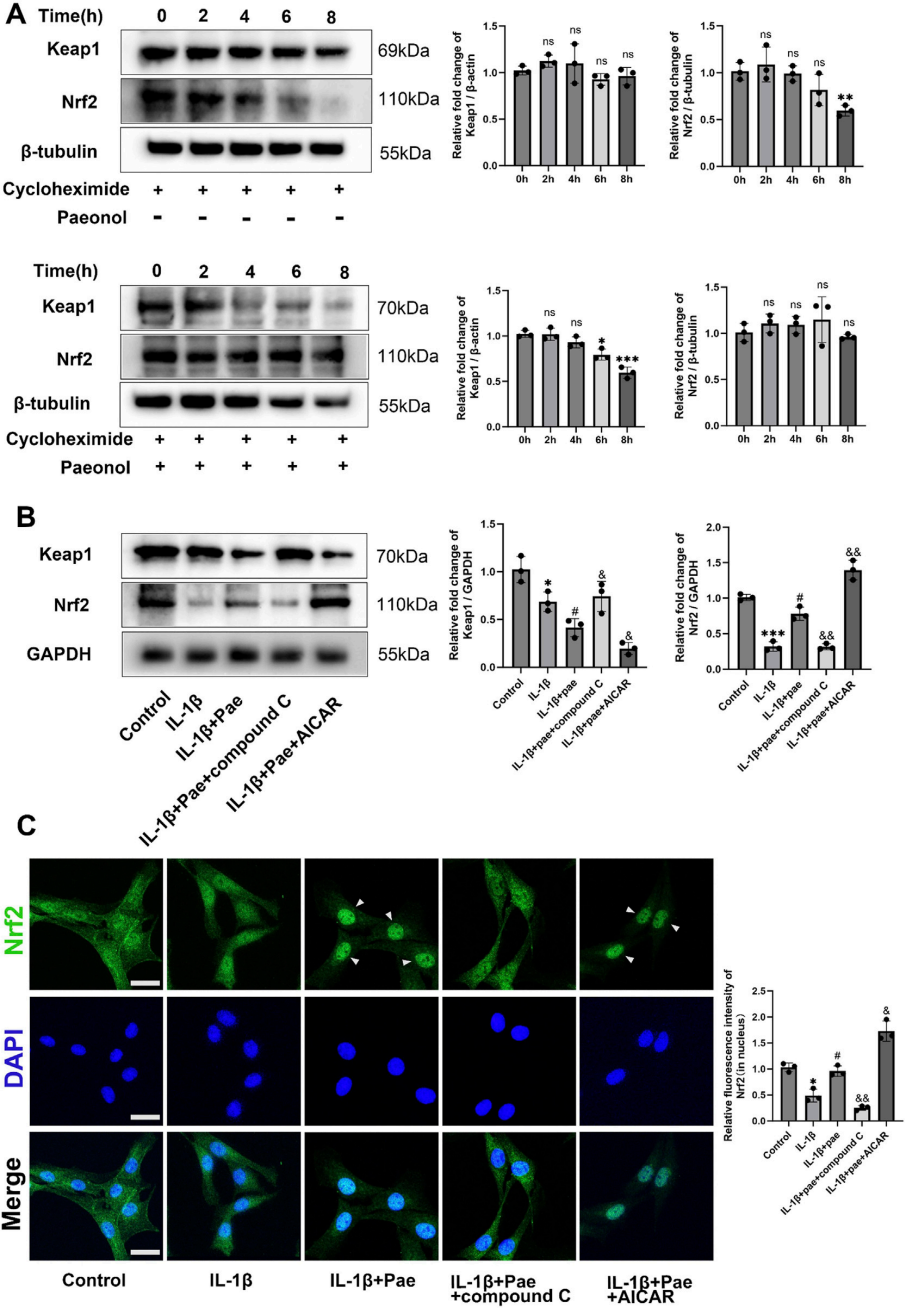
Paeonol protects Nrf2 abundance and promotes Nrf2 nuclear translocation through AMPK in chondrocytes. **(A)** Western blot of Keap1 and Nrf2 in chondrocytes treated with cycloheximide (50 μM) and cycloheximide (50 μM)+ paeonol (10 μM). *P < 0.05, **P < 0.01, ***P < 0.001 versus 0 h group; ns: not significant versus 0 h group. **(B)** Western blot of Keap1 and Nrf2 in chondrocytes treated with paeonol alone, paeonol + AMPK inhibitor compound C and paeonol + AMPK agonist AICAR. **(C)** Immunofluorescence staining for Nrf2 in chondrocyte treated with paeonol, paeonol + compound C, and paeonol + AICAR. Scale bar = 30 μm. Data are means ± SD. (n = 3). *P < 0.05, ***P < *0.001* versus control group; ^*#*^ *P* < *0.05* versus IL-1β group; *&P* < *0.05*, *&&P* < *0.01* versus IL-1β+pae group. Pae, Paeonol.

Nrf2, reported to be downstream of AMPK, is a major antioxidant factor for cells and is involved in a variety of inflammatory responses. In the absence of stress, Nrf2 is retained in the cytoplasm and binds to an E3 ubiquitin ligase substrate adaptor protein [Kelch-like ECH-related protein 1 (Keap1)]. Under oxidative stress, the cysteine residue of Keap1 binds to oxidants and is modified to release Nrf2 into the nucleus for antioxidant defense. We used cycloheximide to inhibit protein synthesis. The results showed that Keap1 expression remained stable, but paeonol induced continuous degradation of Keap1 while maintaining the concentration of Nrf2 (Figure 8A). Inhibition of AMPKα by Compound C lost the ability of paeonol to induce Keap1 degradation and maintain Nrf2 stability. In contrast, AICAR, an AMPK activator, increased the effects of paeonol on keap1 and Nrf2 (Figure 8B). These data suggest that AMPKα is required for paeonol to maintain Nrf2 abundance in chondrocytes by promoting Keap1 degradation. Subsequently, confocal microscopy images showed that paeonol promoted Nrf2 translocation into the nucleus, but this interaction was blocked when AMPK was inhibited in chondrocytes (Figure 8C). This further confirms the important role of AMPK in the paeonol regulation of Nrf2 against oxidative stress.

**FIGURE 9:**
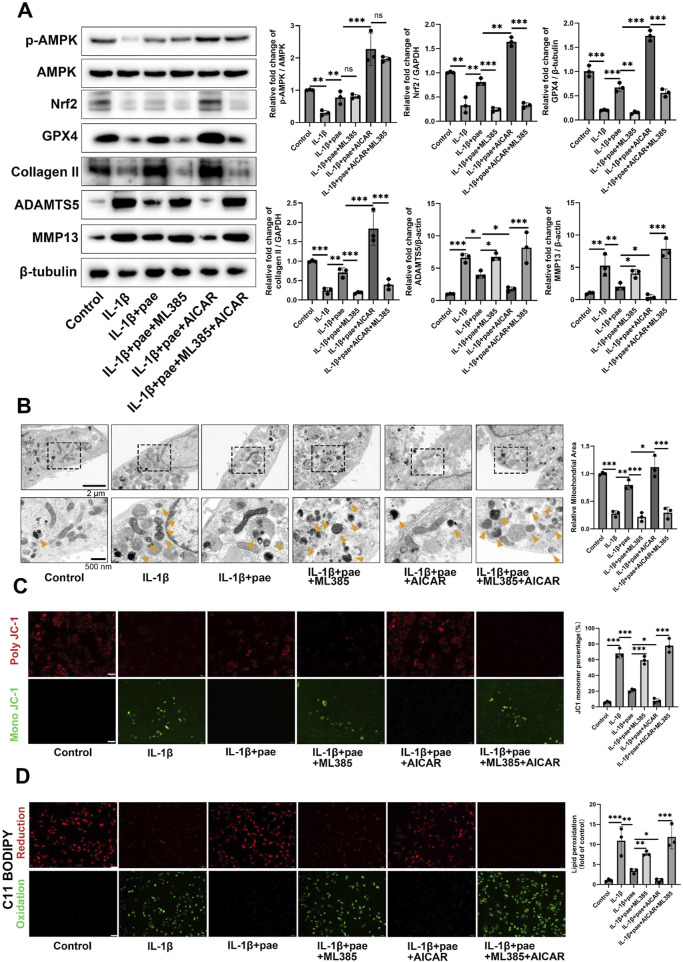
Paeonol regulates GPX4 expression by the AMPK-Nrf2 signaling pathway and further regulates ferroptosis and lipid peroxidation in chondrocytes. **(A)** Western blot of p-AMPK, AMPK, collagen II, inflammation biomarkers ADAMTS 5, MMP13, and ferroptosis biomarkers GPX4 in the presence of paeonol, paeonol + Nrf2 inhibitor ML385, paeonol + AMPK agonist AICAR, and paeonol + ML385+ AICAR. **(B)** Transmission electron microscopy of chondrocytes. Yellow arrows represent damaged mitochondria. **(C)** ΔΨm of chondrocytes in different treatment groups. The JC-1 polymer emits red fluorescence. The decrease in ΔΨm caused the JC-1 monomer to emit green fluorescence. Scale bar = 200 μm. **(D)** Lipid ROS levels in chondrocytes in different treatment groups were detected by C11-BODIPY. Scale bar = 200 μm. *P < 0.05, **P < 0.01, ***P < *0.001*; ns, not significant. Data are means ± SD. (n = 3). Pae, Paeonol.

Then, we further examined the upstream and downstream relationship between AMPK and Nrf2 and its effect on ferroptosis in chondrocytes. Results showed that IL-1β reduced the phosphorylation of AMPK and the expression of Nrf2. However, paeonol alleviated the decline of AMPK and Nrf2. Notely, the addition of Nrf2 inhibitor ML385 did not affect AMPK phosphorylation but significantly inhibited the anti-inflammatory and anti-ferroptosis abilities of paeonol. Specifically, the protein levels of GPX4 and COLII significantly decreased, and MMP13 and ADAMTS5 significantly increased (Figure 9A). The addition of AICAR, an AMPK agonist, significantly increased Nrf2 protein content and enhanced the anti-inflammatory and anti-ferroptosis effects of paeonol. Specifically, the protein levels of GPX4 and COL2 significantly increased, and MMP13 and ADAMTS5 significantly decreased (Figure 9A). More notably, the potent AMPK/paeonol-induced anti-inflammatory and anti-ferroptosis effects were partially blocked by the co-treatment of AMPK agonist and Nrf2 inhibitor, indicating that Nrf2 is a downstream factor of AMPK and both play an important role in paeonol-regulated GPX4-related anti-ferroptosis effect in chondrocytes (Figure 9A). Mitochondrial morphology of chondrocytes was observed by transmission electron microscopy (Figure 9B). The results showed that paeonol significantly alleviated IL-1β-induced mitochondrial damage. Moreover, the ML385, an Nrf2 inhibitor, attenuated the therapeutic effect of paeonol, as evidenced by the reduction of mitochondrial volume and cristae. AICAR, an AMPK agonist, further enhanced the anti-ferroptosis effect of paeonol. Noteworthy, the co-administration of ML385 and AICAR blocked the enhancement effect of AICAR on the anti-ferroptosis of paeonol. This further suggests that Nrf2 is a vital element in the regulation of ferroptosis of chondrocytes by paeonol via the AMPK signal channel. JC1 staining and C11 BODIPY staining confirmed these results. The addition of ML385 and AICAR to paeonol blocked the paeonol anti-mitochondrial membrane damage and anti-lipid peroxidation effect stimulated by AMPK agonists (Figures 9C, D).

## Discussion

Knee osteoarthritis is a chronic disabling arthritis characterized by cartilage degeneration, and effective cartilage repair methods are urgent (Srivastava, 2023; Lv et al., 2022). Therefore, finding new therapeutic targets for KOA will be of great significance for the prevention and treatment of KOA. Ferroptosis, a novel mode of programmed cell death, plays a key role in a variety of pathophysiological processes and is closely related to the pathogenesis of human disease progression, including osteoarthritis (Dixon and Olzmann, 2024; Sun et al., 2021). As a class of natural polyphenols, paeonol exerts certain anti-inflammatory and anti-oxidation abilities (Liu et al., 2017), but its ability to inhibit the ferroptosis of chondrocytes and the mechanism are still unclear. In the present study, we first demonstrated that IL-1β can induce ferroptosis in chondrocytes. Furthermore, we found that paeonol could alleviate chondrocyte inflammatory injury by inhibiting ferroptosis level. Mechanismally, paeonol can activate AMPK-Nrf2-GPX4 signaling pathway to stimulate the endogenous antioxidant system and reduce ferroptosis in chondrocytes. This study suggests that the anti-ferroptosis effect of paeonol is a promising therapeutic strategy for the prevention and treatment of KOA.

In the past 5 years, studies have confirmed that ferroptosis of chondrocytes is closely related to the occurrence and development of KOA. Mechanismally, the breakdown of the GPX4-related anti-lipid peroxidation system, the increase of intracellular Fe^2+^ concentration to promote the Fenton reaction, and the activation of ferroautophagy can lead to ferroptosis of chondrocytes and destroy cartilage homeostasis (Yao et al., 2020; Jing et al., 2021; Sun et al., 2023). Consistent with current findings, our study confirms that IL-1β at 10 ng/mL can induce ferroptosis of chondrocytes, as evidenced by the decrease of GPX4 and SLC7A11 protein levels, suggesting a disorder of the cellular endogenous antioxidant system. Subsequently, IL-1β induced mitochondrial membrane potential damage and increased lipid peroxidation levels in chondrocytes. However, it is worth noting that we did not find that 10 ng/mL IL-1β and different concentrations of paeonol regulated intracellular iron concentration regulatory proteins FTH1 and TfR1, which is inconsistent with the results of Jing et al. (2021). The storage and transport of iron in cells is a complex regulatory system, and FTH and TfR1 are the representative proteins in the process (Sun et al., 2024). However, the maintenance of iron homeostasis in cells is far more than the maintenance of the above two proteins. A variety of proteins, such as transferrin, ferritins, hepcidin, and ferroportin jointly play an important role in maintaining cellular iron homeostasis (Rochette et al., 2022). The diversity and unknown regulation of cellular iron metabolism is the potential reason for the inconsistency between the results of this study and those of Jing et al. (2021). The FerroOrange fluorescent probe staining in the present study confirmed that IL-1β increased the intracellular ferrous ion level in chondrocytes, which was consistent with the current results, but paeonol did not exert the ability to regulate the intracellular ferrous ion level. These results suggest that paeonol exerts an anti-ferroptosis effect partly through anti-lipid peroxidation rather than regulating intracellular iron metabolism.

The AMPK signaling pathway is involved in the development of OA, and the lack of AMPK in chondrocytes aggravates cartilage degeneration in OA mice (Yao et al., 2023). To clarify the potential mechanism of paeonol regulating ferroptosis in chondrocytes, we examined the effects of paeonol on AMPK phosphorylation, ferroptosis, and inflammatory biomarkers. Moreover, the addition of paeonol significantly increased the phosphorylation of AMPK, which was inhibited by IL-1β, and the extra addition of AMPK agonists and inhibitors could stimulate and reverse the anti-ferroptosis effect of paeonol. This is consistent with the current research results. For example, Shen’s study found that paeonol improved hyperlipidemia and autophagy in mice by regulating AMPK/mTOR pathway (Shen et al., 2024), and Choy’s study found that paeonol inhibits endoplasmic reticulum stress-induced endothelial dysfunction through AMPK/PPARdelta signaling pathway (Choy et al., 2016), and Wu’s study found that paeonol upregulates autophagy to inhibit the proliferation of vascular smooth muscle cells through the AMPK/mTOR signaling pathway (Wu et al., 2018). This study is the first to demonstrate the important role of AMPK-related signaling pathway in paeonol-regulated ferroptosis in chondrocytes. However, the effective concentration of paeonol on AMPK regulation in cells is still divergent. For example, Choy’s study found that as little as paeonol 0.1 μM can regulate AMPK phosphorylation in endothelial cells (Choy et al., 2016). Wu’s study found that paeonol 30 μM could regulate AMPK phosphorylation in vascular smooth muscle cells (Wu et al., 2018), while the paeonol intervention concentration of Shen’s study was as high as 308 μM (Shen et al., 2024). This discrepancy may stem from differences in sensitivity due to differences in cell types. In this study, we found that 10 μM paeonol significantly regulated AMPK phosphorylation in chondrocytes.

Nrf2 is considered as a major regulator of antioxidant response because many of its downstream target genes are involved in preventing or correcting intracellular REDOX imbalance and subsequently anti-ferroptosis (Torrente and DeNicola, 2022). More importantly, Nrf2 is one of the downstream factors of AMPK (Yang et al., 2020). In our study, we first confirmed that chondrocytes ferroptosis after IL-1β intervention, which was manifested as decreased nuclear translocation of Nrf2 and decreased degradation of Keap1, a negative regulator of Nrf2. The addition of paeonol significantly increased the nuclear translocation level of Nrf2 and promoted the degradation of Keap1 to exert the anti-ferroptosis function, and this process was closely related to AMPK, because the effect of paeonol regulating Nrf2 was partially blocked by the addition of AMPK inhibitor. Other studies also reported the regulation of Nrf2 by paeonol, and most of them were related to anti-oxidative stress. A study by Gao confirmed that paeonol inhibits foam cell ferroptosis and lipid peroxidation through the Nrf2/GPX4 pathway (Gao et al., 2024). This is similar to the potential mechanism of paeonol regulating ferroptosis in chondrocytes found in the present study. However, Gao’s study suggested that the upstream gene regulating Nrf2 was SIRT1, and this study suggested that AMPK (Gao et al., 2024). In fact, both SIRT1 and AMPK are considered as cellular energy regulators (Cantó et al., 2009). SIRT1 may work with AMPK to regulate Nrf2, but the exact cascade needs to be further studied (Cantó et al., 2009).

However, our study is not without limitations. First, as stated in the previous paragraph, the pathway from AMPK to Nrf2 is complex, and we have targeted only one part of this pathway. Second, paeonol reduced ferroptosis and inflammation in chondrocytes, and AMPK-Nrf2-GPX4 signaling was considered to be the underlying mechanism. However, we cannot exclude that other mechanisms may underlie the anti-ferroptosis function of paeonol. Third, although we demonstrated that paeonol plays a key role in inhibiting ferroptosis in chondrocytes *in vitro*, further *in vivo* studies are needed to confirm this, which is our future research plan.

## Conclusion

Taken together, our study demonstrated for the first time that paeonol ameliorated IL-1β-induced ferroptosis and inflammation in chondrocytes. The molecular mechanism may be related to the AMPK/Nrf2/GPX4 pathway regulating ferroptosis. These results suggest that the anti-ferroptosis effect of paeonol in chondrocytes may be a promising new strategy for the treatment of osteoarthritis.

## Material and method

### Primary chondrocytes isolation

To obtain primary chondrocytes, knee cartilage tissue was aseptically collected from 4-week-old SD rats. The cartilage tissue was minced and digested with 0.2% type II collagenase (Gibco, United States) for 8 h at 37°C (Geng et al., 2024). The tissue was repeatedly blown to disperse by a pipettor, whereafter, the suspension was collected through the cell sieve. Finally, the filtered cell suspension was centrifuged at 1,000 rpm for 3 min to obtain primary chondrocytes.

### Chondrocytes culture

The extracted chondrocytes were treated with 10% fetal bovine serum (FBS; Gibco, United States) and 1% peniculin-streptomycin (Gibco, United States) in DMEM under standard conditions (37°C, 5% CO_2_). The medium was changed every 2 days.

### Cell viability assay

Cell viability was detected by CCK-8 assay kit (Biosharp, China). Briefly, chondrocytes were seeded uniformly into 96-well plates. When the cell density was suitable, the medium was replaced with or without IL-1β (Novoprotein, Suzhou, China) (10 ng/mL) and IL-1β+paeonol (Sigma-Aldrich, MO., United States) (1.25–20 μmol). After a specific intervention for 24 h, a new culture medium containing 10 μL CCK-8 solution was added and incubated for 2 h. Absorbance values at 450 nm were determined using a microplate reader (Thermo Fisher Scientific, United States).

### Reduced glutathione content assay

The ratio of reduced glutathione (GSH) and oxidized glutathione (GSSG) was determined by the GSH and GSSG Assay Kit (Beyotime, China). In brief, chondrocytes were seeded uniformly in six-well plates. When the cell density was suitable, the medium was replaced with or without IL-1β (10 ng/mL), IL-1β + paeonol (5, 10 μmol), or IL-1β + paeonol 10 μmol + erastin 5 μmol. At 24 h, cells were harvested and centrifuged. Subsequently, three times the volume of protein removal reagent was added to the cell precipitate, and the supernatant was rapidly freeze-thaw and obtained by centrifugation. The reaction system was subsequently prepared, including 5 μL of sample, 5 μL of protein removal reagent, 150 μL of total glutathione detection solution, and 50 μL of NADPH. A412 was determined by microplate reader after 5 min incubation at room temperature. In order to detect the GSSG content, the GSH removal assistant solution was added to the sample, and the remaining detection steps were the same as above.

### Malondialdehyde (MDA) content assay

Chondrocytes were seeded uniformly in six-well plates and treated with or without IL-1β 10 ng/mL, IL-1β+ paeonol 5 μmol, IL-1β+ paeonol 10 μmol, and IL-1β+ paeonol 10 μmol +Erastin 5 μmol for 24 hours. Subsequently, the cells were lysed, and the supernatant was taken to detect the protein concentration. The supernatant was mixed with the MDA detection working solution and heated at 100° C for 15 min. After cooling to room temperature, the mixture was centrifuged at 1,000 g for 10 min at room temperature. Two hundred microliters of the mixture were added to a 96-well plate, and absorbance was subsequently measured at 532 nm using a microplate reader. At the same time, standard samples were set up for parallel detection. The MDA content of the samples was calculated.

### Lipid ROS assay

Chondrocytes were seeded uniformly in six-well plates and treated with or without IL-1β 10 ng/mL, IL-1β + paeonol 5 μmol, IL-1β + paeonol 10 μmol, IL-1β + paeonol 10 μmol + Erastin 5 μmol, IL-1β + paeonol 10 μmol + ML385 5 μM, IL-1β + paeonol 10 μmol + AICAR 1 mmol, and IL-1β + paeonol 10 μmol + ML385 5 μmol + AICAR 1 mmol for 24 h. Subsequently, cells were washed with PBS and incubated with 1 mL BODIPY 581/591 C11 staining working solution. The cells were incubated in a cell incubator at 37°C for 30 min. Laser scanning confocal microscope was used to observe and fluorescence quantitative.

### Measurements of mitochondrial membrane potential

The cells were treated as in section “Lipid ROS assay”. At the end of the appropriate intervention, the cells were washed with PBS, and then 1 mL of JC-1 staining working solution was added and thoroughly mixed. The cells were incubated in a cell incubator for 20 min at 37°C. Subsequently, the cells were washed twice with JC-1 staining buffer observed under a laser confocal microscope and quantified by fluorescence.

### Ferro Orange assay

Chondrocytes were seeded uniformly in six-well plates and treated with or without IL-1β 10 ng/mL, IL-1β + paeonol 5 μmol, and IL-1β + paeonol 10 μmol for 24 h. Subsequently, the drug-containing medium was removed and replaced with a conventional medium (89% DMEM +10% FBS +1% Penicillin&Streptomycin). FerroOrange working liquid with a concentration of 1 μmol was added and incubated in a 5% CO_2_ incubator at 37°C (Zou et al., 2024). The cells were observed under a fluorescence microscope immediately after incubation.

### Western blot

The cells were treated as in the section “Lipid ROS assay”. After the intervention, the cells were collected and lysed. After centrifugation, the supernatant of the cells was collected, and the supernatant was mixed into the loading buffer at a ratio of 4:1. The mixture was heated at 100°C for 10 min. The proteins are separated on SDS-polyacrylamide gel and subsequently transferred to the PVDF membrane. The membrane was blocked at 5% BSA for 1 h and then incubated in a solution containing primary antibody at 4°C overnight. The next day, the membrane was washed 3 times in TBST for 10 min each time. It was then incubated with a secondary antibody for 1 h, followed by three more TBST washes, each lasting 10 min. Visualization of the blot was performed using an ECL developer.

The primary antibodies involved in this study were collagen II, ADAMTS 5, GPX4, ACSL4, SLC7A11, FTH1 (Abcam, United Kingdom), p-AMPK, AMPK, Keap1 (Cellsignal, United States), MMP13, Nrf2 (Proteintech, China), TfR1 (Huabio, China), β-tubulin, β-actin, GAPDH (Servicebio, China). Moreover, the second antibody, Goat Anti-Rabbit IgG, was purchased from Abcam.

### Immunofluorescence staining

The cells were uniformly planted in a 24-well plate covered with the crawling slice and treated with or without IL-1β 10 ng/mL, IL-1β + paeonol 5 μmol, IL-1β + paeonol 10 μmol, IL-1β + paeonol 10 μmol + Erastin 5 μmol, IL-1β + paeonol 10 μmol + compound C 20 μmol, and IL-1β + paeonol 10 μmol + AICAR 1 mmol for 24 h.

After the intervention, the medium was removed and washed two times with PBS. The cells were fixed with 4% paraformaldehyde for 10 min at room temperature, then washed 3 times with PBS, and PBS containing 0.25% Triton X-100 was added to penetrate the cell membrane. After the cells were washed with PBS 3 times, PBS containing 3% BSA was added and blocked for 30 min at room temperature. Subsequently, GPX4, Nrf2, and MMP13 antibodies were diluted in PBS-1%BSA solution at a ratio of 1:100. After the blocking solution was removed, the primary antibody was added and incubated at 4°C overnight. The next day, the primary antibody was removed, and cells were washed with PBS - 0.35% Tween 20 3 times. Subsequently, the fluorescent secondary antibodies were diluted in PBS - 1% BSA solution at the ratio of 1: 400, and the secondary antibodies were added and incubated for 1 h away from light at room temperature. After the cells were washed three times, DAPI was added to redyeing the nucleus. It was observed by a confocal laser microscope and quantified by fluorescence.

### Transmission electron microscopy (TEM)

Chondrocytes receiving indicated treatments were collected and fixed with 2.5% glutaraldehyde for 4 h at 4°C and with 1% osmic acid/0.1 M phosphate buffer for 2 h at 20°C. Subsequently, the cells were dehydrated, infiltrated, embedded, sliced, and stained with 2% uranium acetate solution. Finally, the ultrastructure was observed by transmission electron microscopy (HT7700, Hitachi, Japan) at 80 kV (Guo et al., 2022).

### Statistical analysis

Data were analyzed through at least 3 independent experiments and expressed as mean ± standard deviation. GraphPad Prism 9.0 was used for statistical analysis. Multiple group differences were analyzed using one-way ANOVA, and a pairwise comparison was achieved using LSD (Gong et al., 2024). *P* < 0.05 was considered statistically significant.

## Data Availability

The original contributions presented in the study are included in the article/supplementary material, further inquiries can be directed to the corresponding author.

## Abbreviations

AMPK, AMP-activated protein kinase; Nrf2, Nuclear factor (erythroid derived-2)-like 2; GPX4, Glutathione peroxidase 4; IL-1β, Interleukin-1β; Keap1, Kelch-like ECH-associated protein 1; Col-II, Collagen II; GAG, Glycosaminoglycan; SIRT, Sirtuins; ADAMTS, Thrombospondin motifs; ACSL4, Acyl-CoA synthetase long-chain family; SLC7A11, Solute carrier family 7a member 11; FTH1, Ferritin heavy chain; MMP13, Matrix metalloprotease 13; TfR1, Transferrin receptor 1; KOA, Knee osteoarthritis; Pae, Paeonol; mTOR, Mammalian target of rapamycin; PPAR, Peroxisome proliferator-activated receptors; DMEM, Dulbecco’s Modified Eagle Medium; FBS, Fetal bovine serum; PVDF, Polyvinylidene fluoride; ROS, Reactive oxygen species; TEM, Transmission electron microscope; CCK-8, Cell Counting Kit-8; GSH, Glutathione; GSSG, Oxidized glutathione; MDA, Malondialdehyde; PBS, Phosphate buffer solution; BSA, Bull serum albumin; TBST, Tris-buffered saline solution + Tween.

## References

[B1] Arden N. K. Perry T. A. Bannuru R. R. Bruyère O. Cooper C. Haugen I. K. (2021). Non-surgical management of knee osteoarthritis: comparison of ESCEO and OARSI 2019 guidelines. Nat. Rev. Rheumatol. 17, 59–66. 10.1038/s41584-020-00523-9 33116279

[B2] Cantó C. Gerhart-Hines Z. Feige J. N. Lagouge M. Noriega L. Milne J. C. (2009). AMPK regulates energy expenditure by modulating NAD+ metabolism and SIRT1 activity. Nature 458, 1056–1060. 10.1038/nature07813 19262508 PMC3616311

[B3] Choy K. W. Mustafa M. R. Lau Y. S. Liu J. Murugan D. Lau C. W. (2016). Paeonol protects against endoplasmic reticulum stress-induced endothelial dysfunction via AMPK/PPARδ signaling pathway. Biochem. Pharmacol. 116, 51–62. 10.1016/j.bcp.2016.07.013 27449753

[B4] Dixon S. J. Olzmann J. A. (2024). The cell biology of ferroptosis. Nat. Rev. Mol. Cell Biol. 25, 424–442. 10.1038/s41580-024-00703-5 38366038 PMC12187608

[B5] Gao M. Dong L. Yang Y. Yan J. Liang Y. Ma X. (2024). The anti-atherosclerotic effect of Paeonol against the lipid accumulation in macrophage-derived foam cells by inhibiting ferroptosis via the SIRT1/NRF2/GPX4 signaling pathway. Biochem. Biophys. Res. Commun. 708, 149788. 10.1016/j.bbrc.2024.149788 38518720

[B6] Geng N. Fan M. Kuang B. Zhang F. Xian M. Deng L. (2024). 10-hydroxy-2-decenoic acid prevents osteoarthritis by targeting aspartyl β hydroxylase and inhibiting chondrocyte senescence in male mice preclinically. Nat. Commun. 15, 7712. 10.1038/s41467-024-51746-3 39231947 PMC11375154

[B7] Gibbs A. J. Gray B. Wallis J. A. Taylor N. F. Kemp J. L. Hunter D. J. (2023). Recommendations for the management of hip and knee osteoarthritis: a systematic review of clinical practice guidelines. Osteoarthr. Cartil. 31, 1280–1292. 10.1016/j.joca.2023.05.015 37394226

[B8] Gong S. Lang S. Wang Y. Li X. Tian A. Ma J. (2024). pH-responsive mesoporous silica nanoparticles loaded with naringin for targeted osteoclast inhibition and bone regeneration. Int. J. Nanomedicine 24, 6337–6358. 10.2147/IJN.S456545 PMC11213539 38946884

[B9] Guo M. Zhu Y. Shi Y. Meng X. Dong X. Zhang H. (2022). Inhibition of ferroptosis promotes retina ganglion cell survival in experimental optic neuropathies. Redox. Biol. 58, 102541. 10.1016/j.redox.2022.102541 36413918 PMC9679710

[B10] He R. Wei Y. Peng Z. Yang J. Zhou Z. Li A. (2024). α-Ketoglutarate alleviates osteoarthritis by inhibiting ferroptosis via the ETV4/SLC7A11/GPX4 signaling pathway. Cell Mol. Biol. Lett. 29, 88. 10.1186/s11658-024-00605-6 38877424 PMC11177415

[B11] Jing X. Du T. Li T. Yang X. Wang G. Liu X. (2021). The detrimental effect of iron on OA chondrocytes: importance of pro-inflammatory cytokines induced iron influx and oxidative stress. J. Cell Mol. Med. 25, 5671–5680. 10.1111/jcmm.16581 33942503 PMC8184674

[B12] Liu C. Han Y. Gu X. Li M. Du Y. Feng N. (2021). Paeonol promotes Opa1-mediated mitochondrial fusion via activating the CK2α-Stat3 pathway in diabetic cardiomyopathy. Redox. Biol. 46, 102098. 10.1016/j.redox.2021.102098 34418601 PMC8385203

[B13] Liu M. Gao X. Shi M. Li M. Sun T. Wang J. (2024). Paeonol could alleviate diabetes-related spermatogenic dysfunction via SIRT3-dependent redox rebalancing. Clin. Transl. Med. 14, 1585. 10.1002/ctm2.1585 PMC10867591 38356399

[B14] Liu M. Zhong S. Kong R. Shao H. Wang C. Piao H. (2017). Paeonol alleviates interleukin-1β-induced inflammatory responses in chondrocytes during osteoarthritis. Biomed. Pharmacother. 95, 914–921. 10.1016/j.biopha.2017.09.011 28910961

[B15] Lv Z. Han J. Li J. Guo H. Fei Y. Sun Z. (2022). Single cell RNA-seq analysis identifies ferroptotic chondrocyte cluster and reveals TRPV1 as an anti-ferroptotic target in osteoarthritis. EBioMedicine 84, 104258. 10.1016/j.ebiom.2022.104258 36137413 PMC9494174

[B16] Miao Y. Chen Y. Xue F. Liu K. Zhu B. Gao J. (2022). Contribution of ferroptosis and GPX4's dual functions to osteoarthritis progression. EBioMedicine 76, 103847. 10.1016/j.ebiom.2022.103847 35101656 PMC8822178

[B17] Rochette L. Dogon G. Rigal E. Zeller M. Cottin Y. Vergely C. (2022). Lipid peroxidation and iron metabolism: two corner stones in the homeostasis control of ferroptosis. Int. J. Mol. Sci. 24, 449. 10.3390/ijms24010449 36613888 PMC9820499

[B18] Sharma L. (2021). Osteoarthritis of the knee. N. Engl. J. Med. 384, 51–59. 10.1056/NEJMcp1903768 33406330

[B19] Shen B. Wen Y. Li S. Zhou Y. Chen J. Yang J. (2024). Paeonol ameliorates hyperlipidemia and autophagy in mice by regulating Nrf2 and AMPK/mTOR pathways. Phytomedicine 132, 155839. 10.1016/j.phymed.2024.155839 38943694

[B20] Srivastava A. K., (2023). Surgical Management of Osteoarthritis of the Knee Work Group, Staff of the American Academy of Orthopaedic Surgeons. J. Am. Acad. Orthop. Surg. 31, 1211–1220. 10.5435/JAAOS-D-23-00338 37883429

[B21] Sun K. Guo Z. Hou L. Xu J. Du T. Xu T. (2021). Iron homeostasis in arthropathies: from pathogenesis to therapeutic potential. Ageing Res. Rev. 72, 101481. 10.1016/j.arr.2021.101481 34606985

[B22] Sun K. Hou L. Guo Z. Wang G. Guo J. Xu J. (2023). JNK-JUN-NCOA4 axis contributes to chondrocyte ferroptosis and aggravates osteoarthritis via ferritinophagy. Free Radic. Biol. Med. 200, 87–101. 10.1016/j.freeradbiomed.2023.03.008 36907253

[B23] Sun Y. Ren Y. Song L. Y. Wang Y. Y. Li T. G. Wu Y. L. (2024). Targeting iron-metabolism:a potential therapeutic strategy for pulmonary fibrosis. Biomed. Pharmacother. 172, 116270. 10.1016/j.biopha.2024.116270 38364737

[B24] Torrente L. DeNicola G. M. (2022). Targeting NRF2 and its downstream processes: opportunities and challenges. Annu. Rev. Pharmacol. Toxicol. 62, 279–300. 10.1146/annurev-pharmtox-052220-104025 34499527

[B25] Wu H. Song A. Hu W. Dai M. (2018). The anti-atherosclerotic effect of paeonol against vascular smooth muscle cell proliferation by up-regulation of autophagy via the AMPK/mTOR signaling pathway. Front. Pharmacol. 8, 948. 10.3389/fphar.2017.00948 29354055 PMC5758604

[B26] Wu R. Liu Y. Zhang F. Dai S. Xue X. Peng C. (2024). Protective mechanism of Paeonol on central nervous system. Phytother. Res. 38, 470–488. 10.1002/ptr.8049 37872838

[B27] Xu Q. Liu X. Mei L. Wen Q. Chen J. Miao J. (2018). Paeonol reduces the nucleocytoplasmic transportation of HMGB1 by upregulating HDAC3 in LPS-induced RAW264.7 cells. Inflammation 41, 1536–1545. 10.1007/s10753-018-0800-0 29736733

[B28] Yang L. Li X. Jiang A. Li X. Chang W. Chen J. (2020). Metformin alleviates lead-induced mitochondrial fragmentation via AMPK/Nrf2 activation in SH-SY5Y cells. Redox Biol. 36, 101626. 10.1016/j.redox.2020.101626 32863218 PMC7334619

[B29] Yao Q. Wu X. Tao C. Gong W. Chen M. Qu M. (2023). Osteoarthritis: pathogenic signaling pathways and therapeutic targets. Signal Transduct. Target Ther. 8, 56. 10.1038/s41392-023-01330-w 36737426 PMC9898571

[B30] Yao X. Sun K. Yu S. Luo J. Guo J. Lin J. (2020). Chondrocyte ferroptosis contribute to the progression of osteoarthritis. J. Orthop. Transl. 17, 33–43. 10.1016/j.jot.2020.09.006 PMC7750492 33376672

[B31] Zhu S. Wang Z. Liang Q. Zhang Y. Li S. Yang L. (2023). Chinese guidelines for the rehabilitation treatment of knee osteoarthritis: an CSPMR evidence-based practice guideline. J. Evid. Based. Med. 16, 376–393. 10.1111/jebm.12555 37743650

[B32] Zou Y. Yang A. Chen B. Deng X. Xie J. Dai D. (2024). crVDAC3 alleviates ferroptosis by impeding HSPB1 ubiquitination and confers trastuzumab deruxtecan resistance in HER2-low breast cancer. Drug resist. updat. 77, 101126. 10.1016/j.drup.2024.101126 39243601

